# Adipose stem cells from patients with Crohn’s disease show a distinctive DNA methylation pattern

**DOI:** 10.1186/s13148-020-00843-3

**Published:** 2020-04-06

**Authors:** Carolina Serena, Monica Millan, Miriam Ejarque, Alfonso Saera-Vila, Elsa Maymó-Masip, Catalina Núñez-Roa, Diandra Monfort-Ferré, Margarida Terrón-Puig, Michelle Bautista, Margarita Menacho, Marc Martí, Eloy Espin, Joan Vendrell, Sonia Fernández-Veledo

**Affiliations:** 1 Institut d´Investigació Sanitària Pere Virgili, Hospital Universitari Joan XXIII, Dr Mallafré Guasch, 4, 43007 Tarragona, Spain; 2 CIBER de Diabetes y Enfermedades Metabólicas Asociadas (CIBERDEM), Instituto de Salud Carlos III, 28014, Madrid, Spain; 3 Colorectal Surgery Unit, Hospital Universitari Joan XXIII, 43007 Tarragona, Spain; 4 Colorectal Surgery Unit, Hospital Universitari La Fe, Valencia, Spain; 5 Digestive Unit, Hospital Universitari Joan XXIII, 43007 Tarragona, Spain; 6 Colorectal Surgery Unit, General Surgery Service, Hospital Valle de Hebron, Universitat Autonoma de Barcelona, 08035 Barcelona, Spain; 7 Universitat Rovira i Virgili, Tarragona, Spain

**Keywords:** Methylome, Inflammatory bowel disease, Adipose tissue, Epigenetics, Gene expression

## Abstract

**Background:**

Crohn’s disease (CD) is characterized by persistent inflammation and ulceration of the small or large bowel, and expansion of mesenteric adipose tissue, termed creeping fat (CF). We previously demonstrated that human adipose-derived stem cells (hASCs) from CF of patients with CD exhibit dysfunctional phenotypes, including a pro-inflammatory profile, high phagocytic capacity, and weak immunosuppressive properties. Importantly, these phenotypes persist in patients in remission and are found in all adipose depots explored including subcutaneous fat. We hypothesized that changes in hASCs are a consequence of epigenetic modifications.

**Methods:**

We applied epigenome-wide profiling with a methylation array (Illumina EPIC/850k array) and gene expression analysis to explore the impact of CD on the methylation signature of hASCs isolated from the subcutaneous fat of patients with CD and healthy controls (*n* = 7 and 5, respectively; cohort I). Differentially methylated positions (*p* value cutoff < 1 × 10^−4^ and ten or more DMPs per gene) and regions (inclusion threshold 0.2, *p* value cutoff < 1 × 10^−2^ and more than 2 DMRs per gene) were identified using dmpfinder and Bumphunter (minfi), respectively. Changes in the expression of differentially methylated genes in hASCs were validated in a second cohort (*n* = 10/10 inactive and active CD and 10 controls; including patients from cohort I) and also in peripheral blood mononuclear cells (PBMCs) of patients with active/inactive CD and of healthy controls (cohort III; *n* = 30 independent subjects).

**Results:**

We found a distinct DNA methylation landscape in hASCs from patients with CD, leading to changes in the expression of differentially methylated genes involved in immune response, metabolic, cell differentiation, and development processes. Notably, the expression of several of these genes in hASCs and PBMCs such as tumor necrosis factor alpha (TNFA) and PR domain zinc finger protein 16 (PRDM16) were not restored to normal (healthy) levels after disease remission.

**Conclusions:**

hASCs of patients with CD exhibit a unique DNA methylation and gene expression profile, but the expression of several genes are only partially restored in patients with inactive CD, both in hASCs and PBMCs. Understanding how CD shapes the functionality of hASCs is critical for investigating the complex pathophysiology of this disease, as well as for the success of cell-based therapies.

**Graphical abstract:**

Human adipose-stem cells isolated from subcutaneous fat of patients with Crohn’s disease exhibit an altered DNA methylation pattern and gene expression profile compared with those isolated from healthy individuals, with immune system, cell differentiation, metabolic and development processes identified as the main pathways affected. Interestingly, the gene expression of several genes involved in these pathways is only partially restored to control levels in patients with inactive Crohn’s disease, both in human adipose-stem cells and peripheral blood mononuclear cells. Understanding how Crohn’s disease shapes the functionality of human adipose-stem cells is critical for investigating the complex pathophysiology of this disease, as well as for the success of cell-based therapies. 
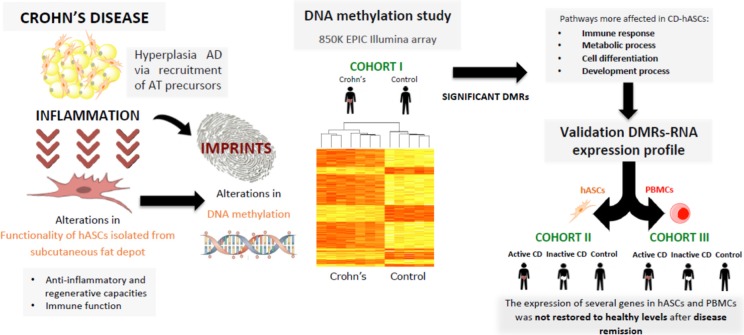

## Background

The prevalence of inflammatory bowel disease (IBD), including Crohn’s disease (CD) and ulcerative colitis, is increasing, especially in western countries, and it is likely to represent an important social and economic burden in the coming years [1, 2]. CD is characterized by persistent inflammation and ulcerations at the small or large bowel, provoking chronic low-grade systemic inflammation and an undulating course of activity, with relapsing cycles occurring after periods of remission. Adipose tissue (AT) is believed to play an active role in the pathogenesis of CD, as the expansion of mesenteric fat attached to the inflamed segments of intestine, also known as “creeping fat,” is a hallmark of the disease that seems to be directly related to disease activity [3–6]. In fact, it has recently been demonstrated that inclusion of the mesentery in ileocolic resection reduces the rate of surgical recurrence [7].

AT is now recognized as a highly active endocrine organ that controls metabolic homeostasis. Emerging evidence points to the immune system as a major contributor to AT homeostasis and function [8]. Indeed, AT contributes importantly to low-grade systemic inflammation in obesity and its comorbidities [9–11]. AT contains an abundant population of multipotent human adipose-derived stem cells (hASCs) that differentiate into several specialized cells, including adipocytes and endothelial cells, and play key roles in immune response [12, 13]. For example, hASCs administered to the circulation have the ability to home to inflamed/injured tissues [14, 15]. Of note, some metabolic stress-induced conditions including CD alter the anti-inflammatory properties of these cells [16–18]. Remarkably, the altered properties of hASCs persist even when patients with CD are in remission, in all AT depots explored including subcutaneous fat [18]. The constancy of this dysfunctional phenotype might reflect changes at the epigenetic level, which ultimately hamper the resolution of the inflammatory events in the sinus of the affected mesenteric AT. In support of this proposition, recent DNA methylation studies in peripheral blood mononuclear cells (PBMCs) revealed that patients with CD have a distinct DNA methylome linked to the expression of differentially methylated genes associated with immune response and inflammation [19–21]. We hypothesized that hASCs from patients with CD are conditioned by a chronic inflammatory milieu, which may alter their DNA methylome and influence their anti-inflammatory and regenerative capacity.

To address this issue, we designed a three-step study: first, we evaluated whether hASCs from the subcutaneous fat of patients with CD have a unique methylation signature that would alter their gene expression profile; second, in a confirmatory study, we performed gene expression analysis in patients considering disease activity; and third, we assessed the reproducibility of the changes in gene expression of candidates genes in an more easily accessible sample, by testing for a specific blood signature.

## Results

### Methylation signature of adipose stem cells from subcutaneous fat in patients with Crohn’s

**Fig. 1:**
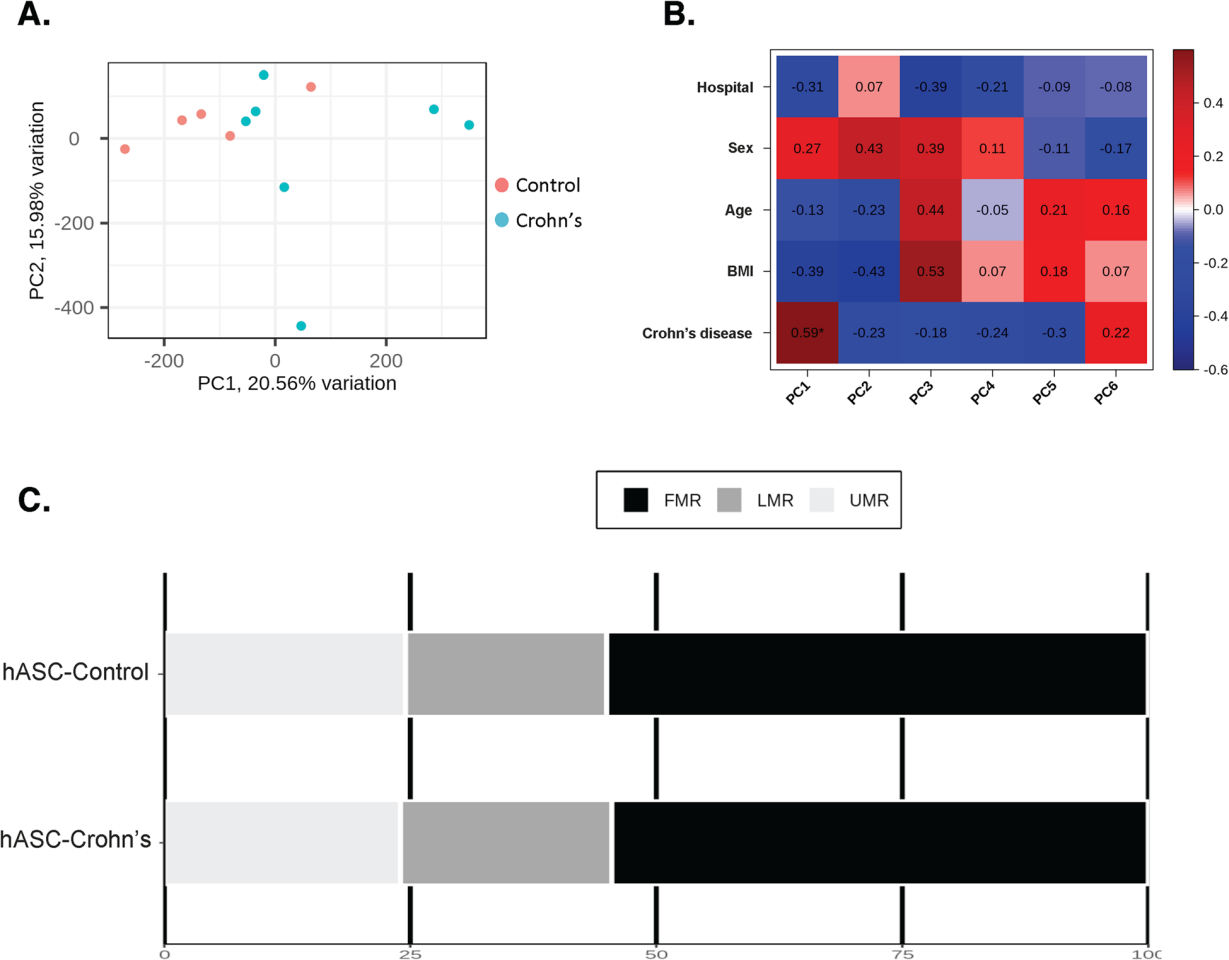
Exploratory data analysis. **a** Score plot of the first two principal components of the overall DNA methylation profiles, showing a discernable differentiation between patients with CD (blue circles) and healthy controls (red circles). **b** Heatmap showing the correlation data between the six main components and clinical parameters such as hospital, sex, age, body mass index [BMI], or disease. Each cell of the heatmap shows the Pearson correlation coefficient. Asterisk indicates significant correlation. **c** Percentage of differentially methylated positions (DMPs) of all the human DNA methylome distributed in three classes: unmethylated regions (UMR < 25% methylated CpGs); low-methylated regions (LMR < 25% methylated CpGs); and fully methylated regions (FMR > 50% methylated CpGs)

We first assessed genome-wide patterns of DNA methylation in hASCs isolated from subcutaneous fat depots of patients with CD and compared them with those from hASCs isolated from healthy controls (*n* = 7 patients with CD and 5 healthy controls; cohort I). No differences in overall mean DNA methylation (calculated by averaging across all probes on the Illumina EPIC/850k array) were observed between the two groups (*p* = 0.33; Student’s *t* test) (data not shown), indicating that CD is not associated with global changes to DNA methylation in this cell type. By contrast, DNA methylation at individual CpG sites showed considerable variability between CD and controls. Principal component analysis (PCA) of the first two principal components (PC1 and PC2) of the methylation data revealed a difference between hASCs of CD and those of controls (Fig. 1a) with PC1 explaining 20.56% of the variance. We also performed a correlation analysis between the confounding variables, hospital, sex, age, and body mass index (BMI) and the principal components (Fig. 1B; data is represented by a heat map). No correlation was found between the six principal components and hospital, sex, age, or BMI, suggesting that these confounding variables did not significantly affect the methylome pattern. Notably, we found a significant correlation between the presence or absence of the disease and PC1 (Fig. 1b).

It is now recognized that, in addition to the expected binary behavior of CpG methylation (either hypo- or hypermethylated), some cytosines show intermediate, yet low, levels of methylation [22]. Thus, we explored potential differences in the segmented methylomes as fully methylated regions (FMRs; > 50% methylated CpGs), low-methylated regions (LMRs; 3.9–50%), and unmethylated regions (UMRs; 1–3.9%), as described [22–26]. The FMR class comprised over 50% of the methylome in all samples (Fig. 1c), and the global percentage of methylation was stable between control- and CD-hASCs. Nevertheless, there was a trend for greater methylation in the LMR region in hASCs isolated from patients with CD relative to those from healthy controls.

### Differentially methylated positions in adipose stem cells isolated from patients with Crohn’s disease

**Fig. 2:**
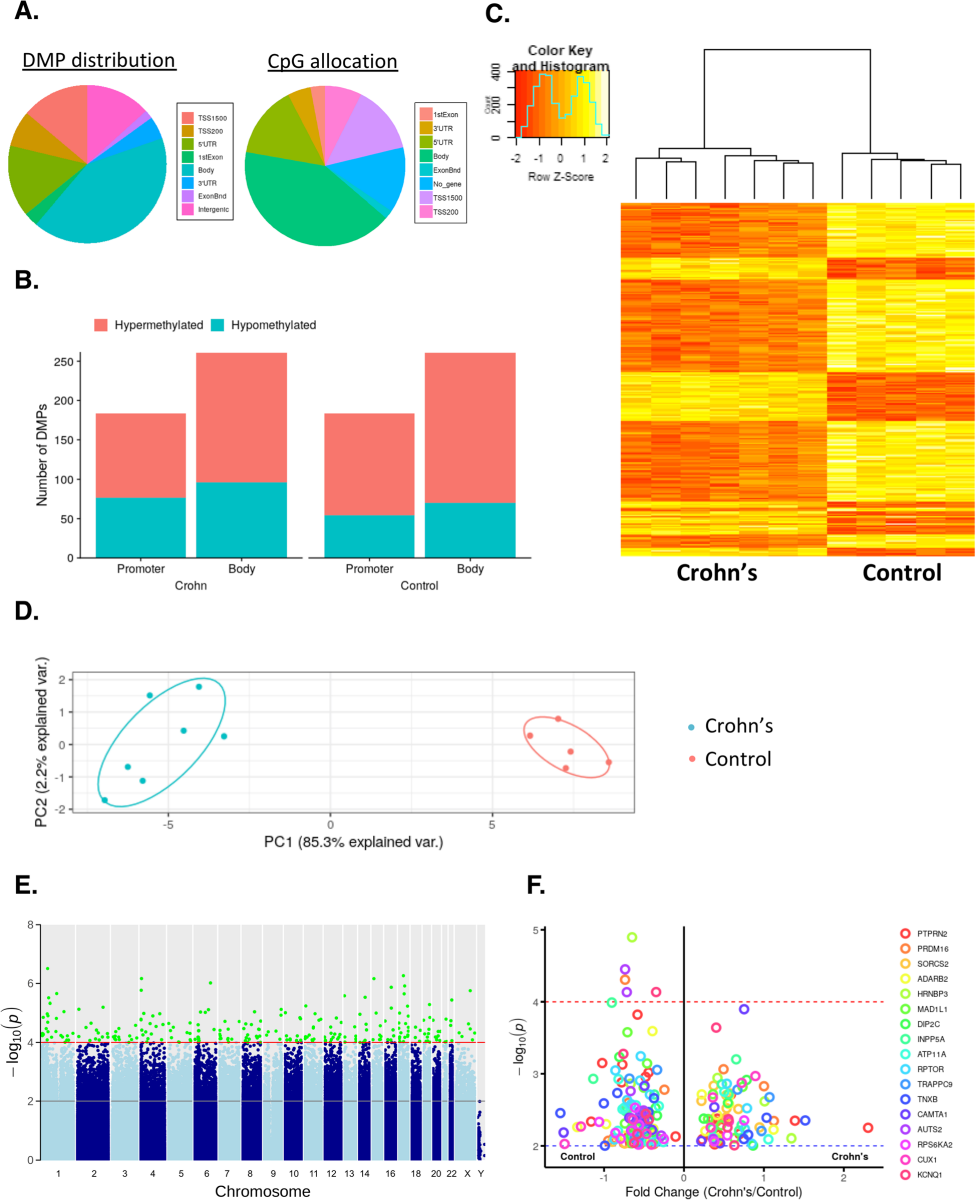
Differentially methylated position analysis in adipose-derived stem cells isolated from patients with Crohn’s disease and healthy controls. **a** Intragenic differentially methylated position (DMP) distribution: approximately 45% of the DMPs are located in the gene body and 35% are located in the promoter. CpG allocation: > 50% of the CpGs are in the open sea region. **b** Distribution of hypo- and hypermethylated DMPs for Crohn’s-hASC and control-hASCs. **c** Heatmap and hierarchical clustering of CpGs according to their methylation profile in Crohn’s disease patients compared with healthy donors. Red bars represent hypomethylated positions and yellow bars hypermethylated positions. **d** Principal component analysis demonstrating the first two main components of the significant CpG data set. **e** Manhattan plot of all DMPs. Significant DMPs are colored in green, achieving the significance threshold (*p* < 0.0001). **f** Volcano plot showing the top DMPs associated with genes. Visualization of the genes with the highest number of significant CpGs. Negative values (points to the left of the graph) indicate hypomethylated; positive values (points to the right of the graph) indicate hypermethylated. ExonBnd, exon boundaries; UTR, untranslated region; TSS, transcription start site; hASCs, human adipose-stem cells

We found statistically significant differences in 18,253 CpGs between CD- and control-hASCs. To specifically evaluate the methylation patterns and the preferential genome location, we segmented all significant differentially methylated positions (DMPs) with a *p* value < 1 × 10^−4^ into the following eight categories according to the Illumina annotations: transcription start site (TSS) 1500, TSS 200 (indicating the number of bases upstream), 5′ untranslated region (UTR), 1st exon, ExonBnd (exon boundary), gene body, 3′ UTR, and no gene location (Fig. 2a, left panel). The DMP distribution within a CpG island is shown in Fig. 2a (right panel), with the majority located in the *open sea* region (> 50%). We found that 35% of methylation sites occurred in the promoter region (including TSS 1500, TSS 200, and 5′ UTR), which is related to gene silencing [23, 27] (Fig. 2b). Of note, most of the methylation sites were found in the gene body (45%) (Fig. 2b), which is known to have a positive correlation with gene transcription [23, 28].

We next performed a heatmap analysis of the significant DMPs between the two groups, which revealed that the DMPs segregated into two clusters (Fig. 2c). We performed a second PCA using only the significant DMPs between hASCs from CD and controls, finding that the explained variance increased considerably when compared with the first PCA (Fig. 2d), with PC1 accounting for 85.3% of the variance. Additionally, we generated a Manhattan plot to observe, at the chromosomal level, where the significant DMPs were located. The results of this analysis indicated that all of the DMPs were evenly distributed throughout the chromosomal complement (Fig. 2e).

Finally, we assigned DMPs found in hASCs between CD and control individuals to candidate genes, which were identified based on a nominal *p* value cutoff of < 1 × 10^−4^ and ten or more DMPs per gene (Supplementary File 1). As illustrated in the volcano plot in Fig. 2f, 17 genes showed significant changes in DMPs: *PTPRN2* (with 28 significant CpGs); *PRDM16* (with 17 significant CpGs); *SORC2*, *ADARB2*, and *HRNBP3* (with 16 significant CpGs); *MAD1L1* (with 14 significant CpGs); *DIPC2* and *INPP5A* (with 13 significant CpGs), *ATP11A*, and *RPTOR* (with 12 significant CpGs); *TRAPPC9*, *TNBX*, *CAMTA1*, *AUST2*, and *RPS6KA2* (with 11 significant CpGs); and *CUX1* and *KCNQ1* (with 10 significant CpGs).

### Differentially methylated regions in hASCs isolated from patients with Crohn’s disease

**Fig. 3:**
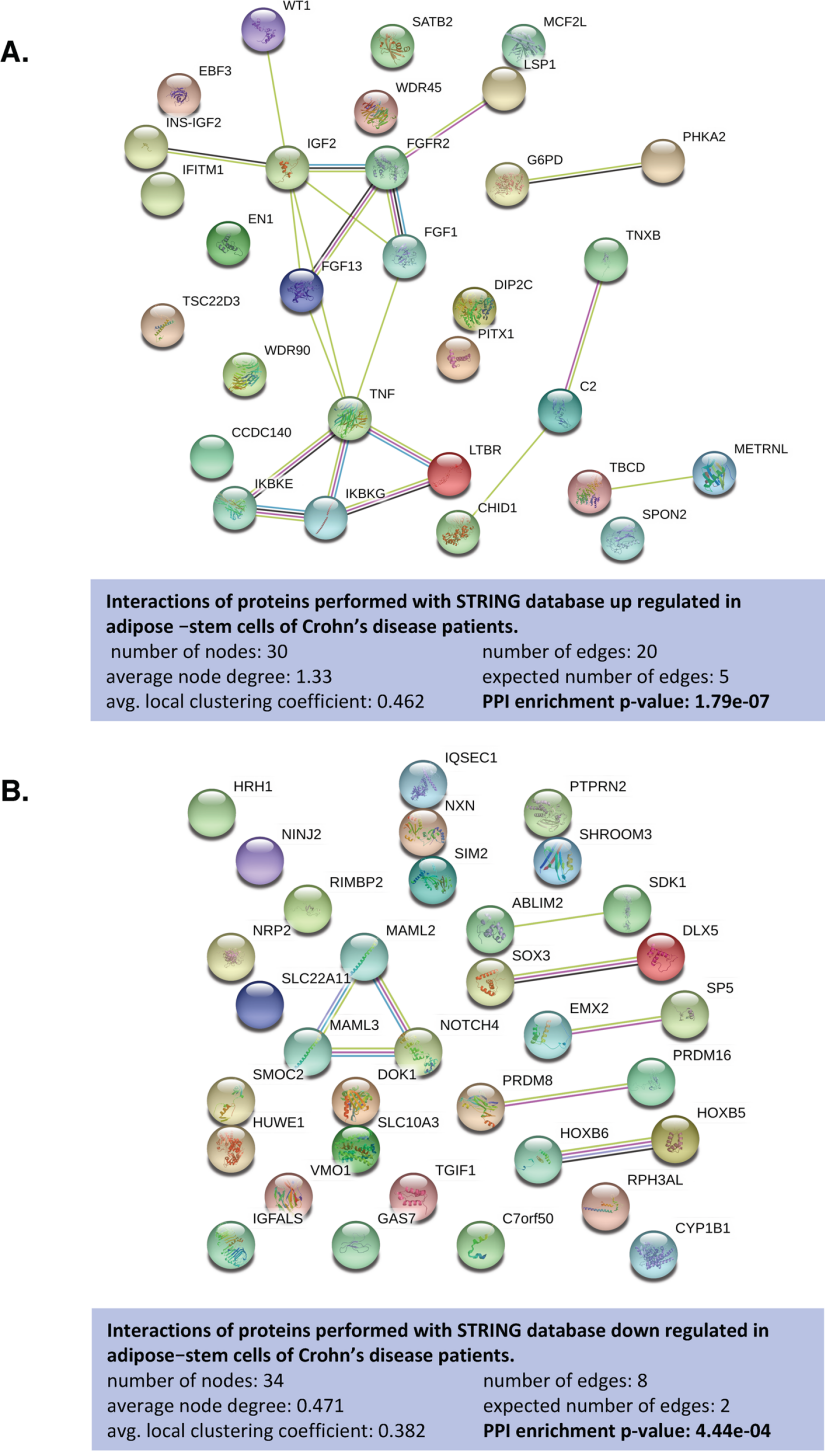
Functional analysis of gene-associated differentially methylated regions in Crohn’s disease. **a** Network of the 30 genes upregulated in Crohn’s disease. **b** Network of the 34 genes downregulated in Crohn’s disease. Such enrichment indicates that the proteins are at least partially biologically connected as a group

Differentially methylated regions (DMRs) were located using the minfi function Bumphunter, using default settings and the smoothing option. Bump hunting with smoothing is a useful methodology for finding regions of biological interest in the context of DNA methylation studies [29]. From our DMR analysis, we found 2150 regions located near known annotated genes (Supplementary File 2). Including those genes with two or more significant DMRs and a *p* value cutoff of < 1 × 10^−2^, we obtained 158 regions annotated with known genes. From these, we specifically selected two or more significant DMRs annotated with the same gene that showed the same direction of effect and were located in the promoter or body regions. We discriminated 37 and 35 genes that we defined as up- or downregulated, respectively, in hASCs of patients with CD. To address the molecular and functional pathways implicated for these genes, we performed functional analysis using STRING v.11.00 (https://string-db.org/). Interestingly, the network analysis of upregulated genes in CD-hASCs revealed a significant protein-protein interaction (PPI) enrichment of the network with a *p* value of 1.79 × 10^−7^, indicating that the proteins were at least partially biologically connected as a group. We selected the more enriched pathways, which formed three categories: immune system, metabolic process, and cell differentiation (Fig. 3a and Table 1). Immune system included 21 enriched gene ontology (GO) terms with a false discovery rate (FDR) ranging from 0.0229 to 0.0484; metabolic process included 7 enriched GO terms with an FDR ranging from 0.0229 to 0.0499; and cell differentiation included 5 enriched GO terms with an FDR ranging from 0.0229 to 0.0358. These categories were confirmed using REVIGO (http://revigo.irb.hr/), which summarizes and visualizes lists of GO terms (Supplementary Figure S1). Of note, REVIGO highlighted immune system process as the most important category affecting hASCs of patients with CD.

**Table 1:**
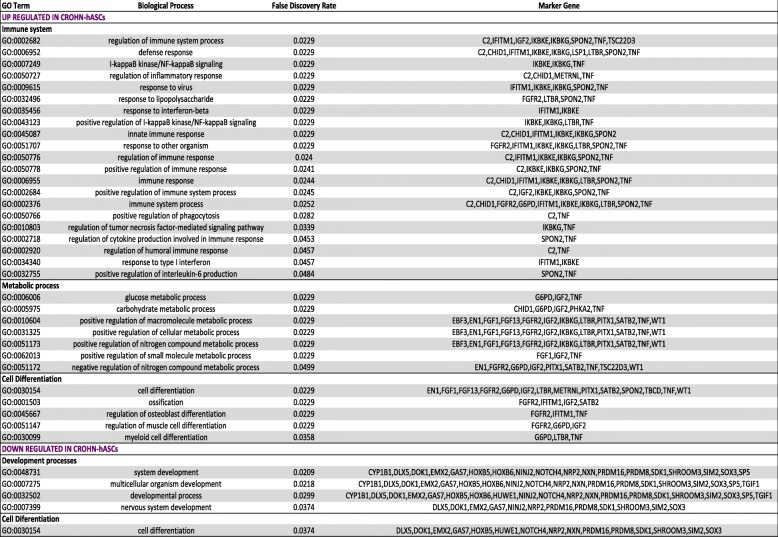
Significantly enriched GO terms of biological processes for genes identified as up- or downregulated in hASCs from patients with Crohn’s disease

The GO analysis was performed in the STRING database website (http://www. string-db.org)

The network analysis of downregulated genes in CD-hASCs revealed a significant PPI enrichment of the network with a *p* value of 4.44 × 10^−4^ (Fig. 3b). The more enriched pathways were development process (included 4 enriched GO terms with an FDR ranging from 0.0209 to 0.0374) and cell differentiation (included 1 enriched GO term with an FDR of 0.0374) (Table 1), which were confirmed using REVIGO (Supplementary Figure S2). The most enriched GO terms of each category as well as the related genes are described in Table 1.

### Confirmatory gene expression analysis in patients with Crohn’s disease with regard to disease activity status

Because DNA methylation can affect transcription, we examined the mRNA expression of a representative selection of at least 3–4 genes per category of identified genes both in patients, according to CD activity, and in healthy subjects, using samples from cohort II (Table 1). To confirm CD activity, we calculated the Crohn’s disease activity index (CDAI) score and assessed clinical and biological parameters that included PCR and fecal calprotectin determinations. Also, an endoscopy evaluation was performed in 80% of the patients (Supplementary Table S2).

#### Immune system

**Fig. 4:**
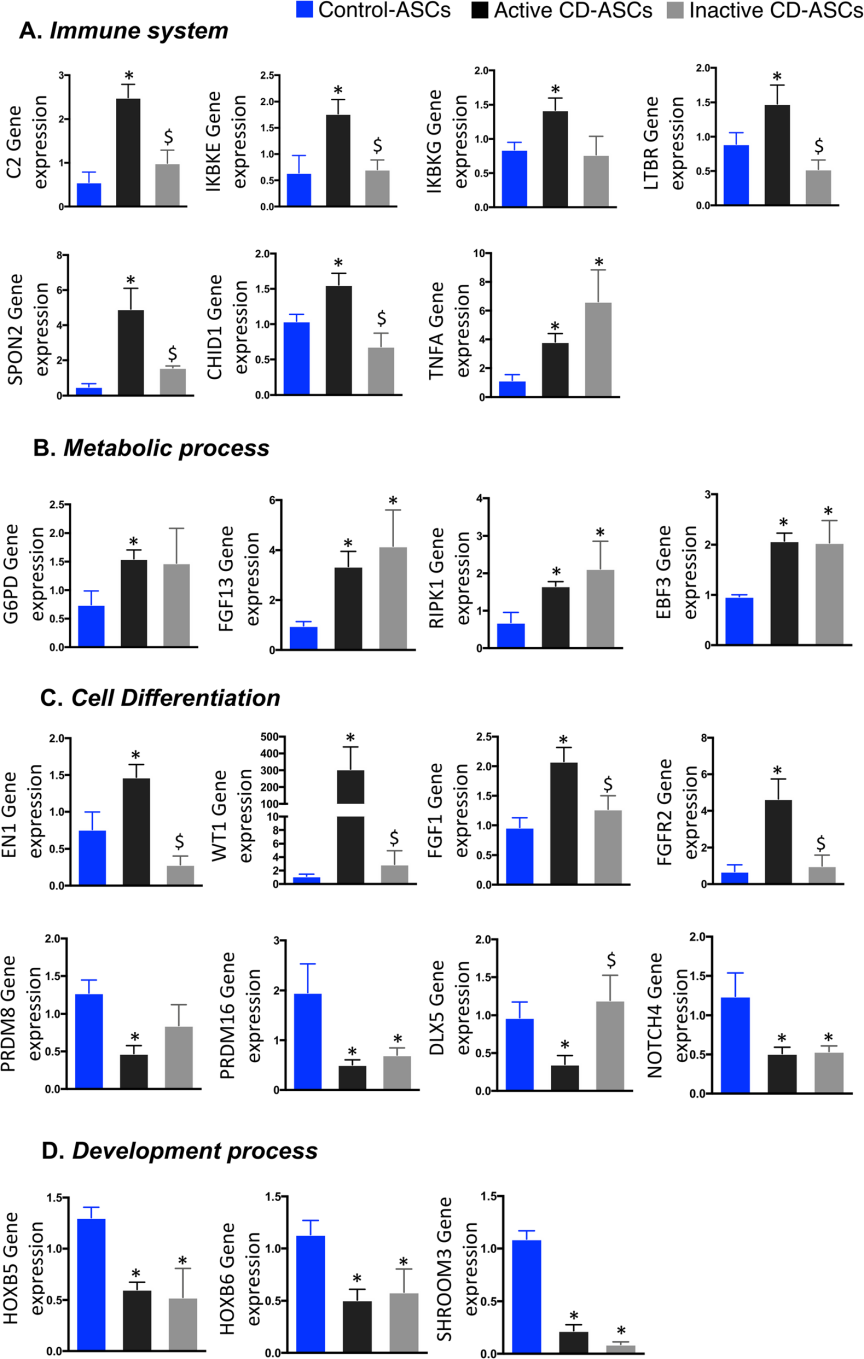
Gene expression of candidate genes obtained from differentially methylated regions between hASCs isolated from patients with active or inactive Crohn’s disease and control individuals. **a** Genes involved in immune system response: *C2*, *IKBKE*, *IKBKG*, *LTBR*, *SPON2*, *CHID1*, and *TNFA*. **b** Genes related to the regulation of metabolism: *G6PD*, *FGF13*, *EBF3*, and *RIPK1*. **c** Genes related to cell differentiation: *EN1*, *WT1*, *FGF1*, *FGFR2*, *PRDM8*, *PRDM16*, and *DLX5*. **d** Genes involved in development process: *HOXB5*, *HOXB6* and *SHROOM3*. One-way analysis of variance with multiple comparisons corrected by Newman-Keuls test was used. *p < 0.05 *versus* control-hASCs; ^$^*p* < 0.05 *versus* active CD-hASCs

The expression of immune system-related genes such as complement 2 (*C2*), inhibitor of nuclear factor kappa B kinase subunit epsilon (*IKBKE*), inhibitor of nuclear factor kappa B kinase subunit beta (*IKBKB*), lymphotoxin beta receptor (*LTBR*), spondin 2 (*SPON2*), chitinase domain-containing 1 (*CHID1*), and tumor necrosis factor alpha (*TNFA*) was significantly higher in hASCs isolated from patients with active disease than in equivalent cells from controls (Fig. 4a). The results were in agreement with the methylation data, and we found significant correlations between gene expression and methylation in some of these genes (Supplementary Figure S3). We observed that the expression of almost all of the genes tested was normalized to control levels in hASCs isolated from patients with inactive disease, indicating that immune system genes affected by methylation marks were for the most part partially restored in patients during episodes of remission. The exception to this was *TNFA*, whose expression remained high in patients during remission, which is in agreement with previous data [18].

#### Metabolic process

Gene expression analysis of metabolic process-related genes is shown in Fig. 4b. Glucose-6-phosphate dehydrogenase (*G6PD*), receptor interacting serine/threonine kinase 1 (*RIPK1*), fibroblast growth factor 13 (*FGF13*), and early B cell factor 3 (*EBF3*) were upregulated in hASCs of patients with active CD relative to controls, as anticipated by the methylation data. Of note, the expression of these genes remained high in hASCs from patients with inactive disease. This indicates that metabolic changes are maintained in periods of remission and suggests that the global inflammatory environment during disease activity shapes the methylome signature in these cells to maintain a high metabolic activity independently of the clinical status of the patient, as we alluded to in an earlier study [18].

#### Cell differentiation process

Gene expression analysis of cell differentiation-related genes is shown in Fig. 4C. Genes upregulated in hASCs from patients with active disease relative to controls included engrailed homeobox 1 (*EN1*), Wilms’ tumor 1 (*WT1*), fibroblast growth factor 1 (*FGF1*), and fibroblast growth factor receptor 2 (*FGFR2*). The gene expression of these cell differentiation markers in hASCs was normalized to control levels in patients with inactive disease. We also found genes related to cell differentiation that were downregulated in hASCs from patients with active disease, including the PR domain zinc finger protein 8 (*PRDM8*), PR domain zinc finger protein 16 (*PRDM16*), distal-less homeobox 5 (*DLX5*), and notch receptor 4 (*NOTCH4*). The gene expression of these cell differentiation markers in hASCs was not normalized to control levels in patients with inactive disease with the exception of *DLX5*, a gene implicated in osteoblastic differentiation in mesenchymal stem cells [30].

#### Development process

We also studied the expression of genes related to development process, such as the homeobox B5 (*HOXB5*), homeobox B6 (*HOXB6*), and shroom family member 3 (*SHROOM3*), finding that they were all significantly downregulated in hASCs from patients with active or inactive disease compared with controls (Fig. 4d). This suggests that epigenetic modifications in these regions remain across CD progression.

### Reproducibility of the observed epigenetic changes in peripheral blood

**Fig. 5:**
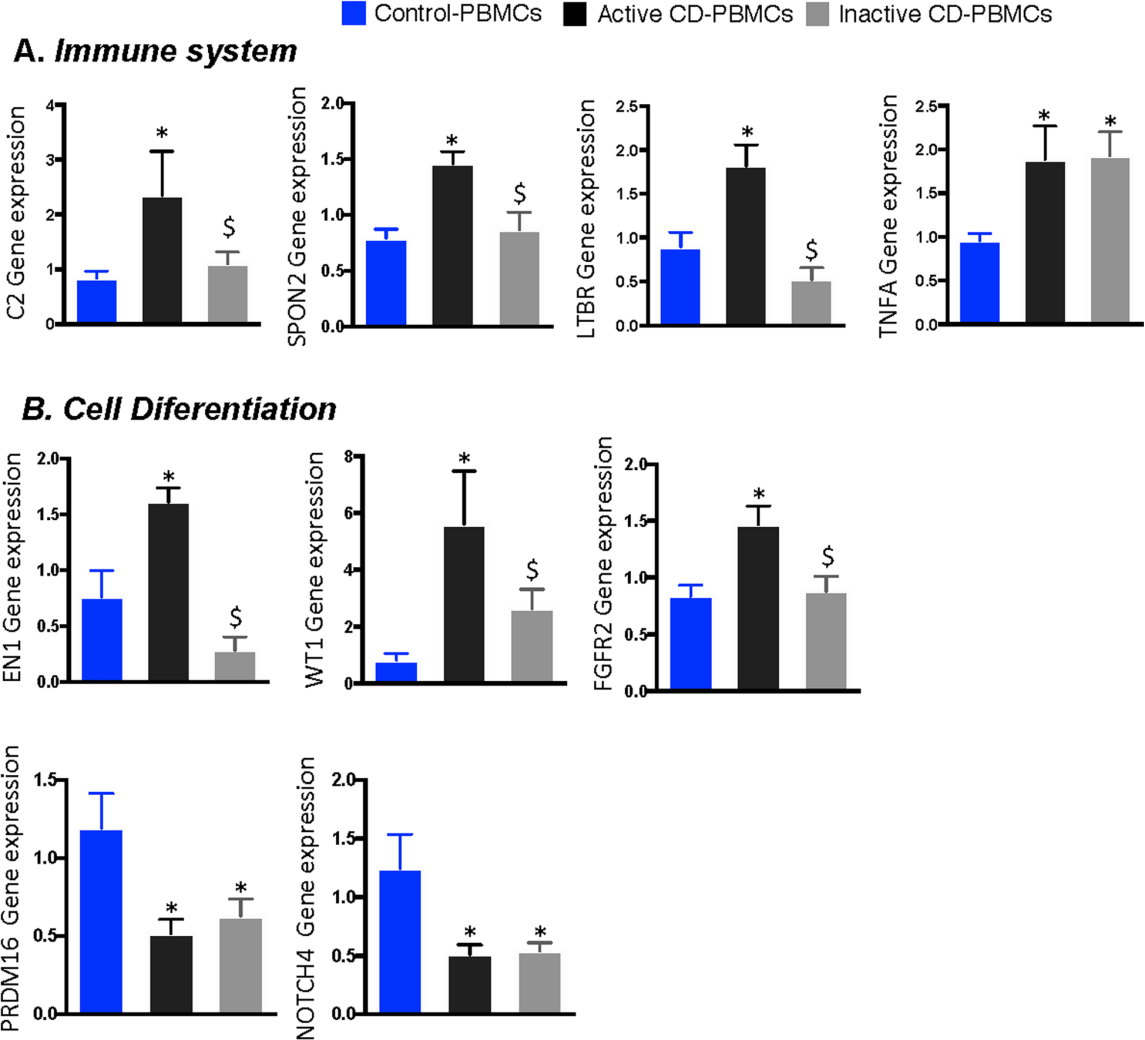
Gene expression of candidate genes obtained from differentially methylated regions between PBMCs isolated from patients with active or inactive Crohn’s disease and control individuals. **a** Genes involved in immune system response: *C2*, *SPON2*, *LTBR*, and *TNFA*. **b** Genes related to cell differentiation: *EN1*, *WT1*, *FGFR2*, and *PRDM16*. One-way analysis of variance with multiple comparisons corrected by Newman-Keuls test was used. *p < 0.05 *versus* control-PBMCs; ^$^*p* < 0.05 *versus* active CD-PBMCs

Lastly, we examined whether the specific epigenetic signatures in hASCs were reproduced in a more easily accessible sample from the patients’ peripheral blood. Accordingly, we tested the expression of candidate genes in cohort III (PBMC cohort isolated from three groups: *n* = 10/10 active/inactive CD and *n* = 10 controls), to validate the changes in gene expression observed in hASCs between healthy controls and patients with CD. Figure 5 shows the expression data for the 9 genes in which we observed significant variability in hASCs. We found that the genes that showed the same behavior in PBMCs and hASCs were those related to immune system and cell differentiation. Accordingly, PBMC expression of the immune system-related genes *C2*, *SPON2*, and *LTBR* was normalized in patients with inactive CD, whereas *TNFA* expression was not (Fig. 5a). Similarly, PBMC expression of cell differentiation-related genes *EN1*, *WT1*, and *FGFR2* was at control levels in patients with inactive disease with the exception of *PRDM16*, which was not significantly recovered in these patients (Fig. 5b). No significant differences were found in gene expression for the other genes (*IKBKE*, *IKBKG*, *CHID1*, *G6PD*, *RIPK1*, *FGF13*, *EBF3*, *FGF1*, *PRDM8*, *DLX5*) (data not shown).

## Discussion

We previously reported that hASCs isolated from the subcutaneous adipose tissue (SAT) of patients with CD mimic some of the features of equivalent cells from the CF of the affected colonic tissue, including functional defects [18]. Here, we report a specific epigenetic DNA methylation signature in hASCs from the SAT of patients with CD. Moreover, we show that changes to DNA methylation-related gene expression both in hASCs and PBMCs are only partially restored in patients with inactive CD, overall suggesting the persistence of latent epigenetic modifications involving several genes mainly related to immune system and metabolic and cell differentiation processes.

### Differentially methylated positions in patients with Crohn’s disease

The examination of the chromosomal distribution of DMPs in hASCs between patients with CD and healthy controls revealed no significant differences; however, our analysis yielded 17 associated genes with more than ten CpG sites significantly differentially methylated. The two most significant DMPs were found in *PTPRN2* and *PRMD16*. *PTPRN2* encodes a protein tyrosine phosphatase implicated in various biological processes including tumor pathogenesis and autoimmune disease [31–34]. Supporting our present findings, *PTPRN2* has previously been reported to be the most significant DMP in PBMCs from patients with CD [19]. Interestingly, *PTPRN2* shows dysregulated DNA methylation in obese children [31], pointing to a link between metabolic dysfunction and immune response. Indeed, specific protein tyrosine phosphatases associated with autoimmune diseases have been detected in rat gastrointestinal endocrine cells [35]. Our finding of a dysregulation of DNA methylation in *PTPRN2* in patients with CD (Fig. 2e, Supplementary Figure S4) suggests that this region could be a hot-spot risk marker that deserves further study. This change in DNA methylation was, nevertheless, not reflected by significant changes in gene expression (although there was a trend for downregulation) (Supplementary Figure S4), possibly because of the small sample size used. *PRMD16* is a transcription factor that activates brown fat-specific genes and is also associated with insulin sensitivity in obese subjects, linking adipose tissue expression and systemic metabolic homeostasis [36]. The gene expression analysis revealed significantly lower expression of *PRDM16* both in hASCs and PBMCs from patients relative to controls. The downregulation of *PRDM16* and *PTPNR2* expression was independent of the clinical status of CD, suggesting a persistence of the epigenetic changes in patients with CD despite a diagnosis of clinical remission. Overall, it is tempting to speculate on a putative link between CD and dysmetabolism mediated by AT.

### Differentially methylated regions in Crohn’s disease

The analysis of DMRs in hASCs identified four major categories: *immune system*, *metabolic*, *cell differentiation*, and *development processes*. Examination of the expression of the most representative genes in these categories highlighted a concurrence with the methylation data in patients from the discovery cohort I. Accordingly, we confirmed an increase in pro-inflammatory markers such as *SPON2*, which is highly expressed both in colon and in adipose tissue and promotes M1-like macrophage recruitment [37]; *C2*, which activates complement and triggers inflammation [38]; *IKBKE* and *IKBKB*, key drivers of the NF-kappa-B inflammatory pathway [39]; *CH1D1*, which has been recently reported to have high expression in PBMCs from patients with rheumatoid arthritis [40], functioning as a regulator of the inflammatory response by macrophages; and *TNFA*, a gene involved in the regulation of a wide spectrum of biological processes and implicated in a variety of diseases, including autoimmune disease and insulin resistance, among others [41–43]. The gene expression of the tested immune system genes was normalized in hASCs of patients in disease remission with the exception of *TNFA*, which remained highly expressed even in the inactive period of CD. These results are in agreement with our previous study showing that *TNFA* gene expression in hASCs remains high during CD relapse [18] and suggest that epigenetic modifications might induce the persistence of a latent inflammation in a background of apparent clinical remission.

Genes related to different *metabolic process* were also modified. We found a significant increase in the expression of genes involved in several metabolic intra-cellular pathways (enzymes, kinases, and growth factors, etc.) in hASCs from patients with active disease, and high gene expression levels were also preserved during the inactive disease period, indicating a persistence of metabolic disturbances.

Regarding markers of *cell differentiation* processes in hASCs, we observed a dual expression pattern: an increase in genes mostly related to adipogenesis and/or osteoblast differentiation (*EN1*, *WT1*, *FGF1*, and *FGFR2*) and a decrease in transcription factors relevant to cell differentiation and maturation (*PRDM8*, *PRDM16*, and *DLX5*) with a return to healthy control levels during CD remission (with the exception of *PRDM16*, as described above).

Finally, genes involved in *development processes* were downregulated in CD-hASCs. Again, disease remission was not accompanied by the restoration of mRNA expression of the studied genes. HOX genes are among the best studied genes in developmental biology and are responsible for driving the differentiation of tissue stem cells along their respective lineages to maintain the correct function of tissues and organs [44]. Thus, downregulation of these genes might indicate an alteration in the stemness function of hASCs in patients with CD, which could help to explain some of the functional alterations (loss of anti-inflammatory and regenerative capacity) previously described [18].

### Verification of candidate genes in circulating PBMCs in patients with Crohn’s disease

Modifications in the gene expression profile observed in hASCs according to the methylation pattern were also explored in PBMCs, as they represent a more accessible source for sampling. Also, epigenetic modifications to hASCs might be reflected in peripheral blood, as already demonstrated in other inflammatory diseases such as obesity [45]. Indeed, changes to the PBMC methylome in CD have previously been described, revealing that differentially methylated genes implicated in immune response are the most frequently affected [19, 46]. In agreement with these reports, we found the same enriched GO terms in hASCs in CD such as immune response and immune system process, and we found that some of the changes found in hASCs were replicated in PBMCs, even when patients were classified according to clinical status.

Globally, we found that alterations in DNA methylation of hASCs in CD patients affect mainly immune system, cell differentiation, metabolic, and development processes and that changes to DNA methylation correlate with changes in gene expression. Some of these alterations were maintained despite the clinical remission of the disease. These observations may have important repercussions in the clinical setting, in particular the use of hASCs for autologous transplantation in some complications of CD, considering their immune-suppressive and regenerative capabilities [47–49].

This study has some limitations that warrant discussion. It is an observational study, with a limited number of cases, and it would be necessary to monitor the evolution of the patients to evaluate the repercussions of the methylation changes for the natural history of the disease. Larger prospective studies in patients will be necessary to decipher the clinical relevance of these epigenetic changes.

Overall, our study has several strengths. First, we present a specific methylome signature in adipocyte precursors from the SAT of patients with CD, including significant DMRs. Second, we show that these epigenetic changes translate into apparent changes in the mRNA expression of several genes, with a special bearing on inflammatory pathways, which are only partially reversed during remission periods of the disease. Finally, we demonstrate that these changes are replicated in PBMCs, pointing to a subtle persistence of the epigenetic changes observed in active disease despite a clinical diagnosis of remission.

## Conclusions

Adipose-stem cells isolated from patients with CD exhibit altered DNA methylation and gene expression profiles, with immune system, cell differentiation, metabolic, and development processes identified as the main pathways affected. Interestingly, the gene expression of several genes involved in these pathways is only partially restored in patients with inactive CD, both in hASCs and PBMCs. Understanding how CD shapes the functionality of hASCs is critical for investigating the complex pathophysiology of this disease, as well as for the success of cell-based therapies.

## Methods

### Study population

Patients and controls were recruited at the University Hospital Joan XXIII (Tarragona, Spain) and University Hospital Vall d’Hebrón (Barcelona, Spain) in accordance with the tenets of the Declaration of Helsinki. The corresponding hospital ethics committees approved the study and written informed consent was obtained from all participants before entering the study. Donors were classified as those in relapse (active) or in remission (inactive) according to the CDAI score as well as biological and clinical parameters such as PCR and fecal calprotectin. Furthermore, endoscopic evaluation was performed in the majority of patients [20, 21, 50, 51]. Healthy subjects and donors with inactive CD were recruited from age- and gender-matched subjects undergoing non-acute surgical interventions such as hernia or cholecystectomy, in a scheduled routine surgery. Patients with active CD were recruited from those undergoing surgery for symptomatic complications. The following three cohorts were included in the study: *cohort I—methylation study*. hASCs were isolated from SAT biopsies of 7 patients with active CD and 5 healthy controls; 71% of patients had endoscopic evaluation. *Cohort II—gene expression study*. The number of subjects included in the first cohort was increased to 10. Accordingly, hASCs were isolated from SAT biopsies of 10 active and 10 inactive patients with CD and 10 healthy controls. Of note, the correlation studies between methylation and gene expression data were only performed between data from the same subject; 80% of CD patients had endoscopic evaluation. *Cohort III—PBMC study cohort*. Thirty new subjects were enrolled in this cohort. PBMCs were isolated from 20 patients with CD (10 active and 10 inactive) and 10 healthy controls. Biochemical and anthropometric variables of cohorts I, II, and II are presented in Supplementary Tables S1, S2, and S3, respectively.

### Adipose stem cell isolation and culture

hASCs were isolated as described [52, 53]. Briefly, SAT was washed extensively with phosphate buffered saline (PBS) to remove debris and then treated with 0.1% collagenase in PBS-1% BSA for 1 h at 37 °C with gentle agitation. Digested samples were centrifuged at 300 × *g* at 4 °C for 5 min to separate adipocytes from stromal cells. The cell pellet containing the stromal fraction was re-suspended in stromal culture medium consisting of DMEM/F12 medium, 10% fetal bovine serum, and 1% antibiotic/antimycotic solution. To prevent spontaneous differentiation, primary cultures of hASCs at passage 0 (P0) were grown to 90% confluence and harvested with trypsin-EDTA, and then aliquots (1 × 10^6^ cells) were cryopreserved in liquid nitrogen until required [54].

### Adipose stem cell immunophenotyping

Cells (2 × 10^5^) were incubated with a panel of primary antibodies (described in Supplementary Table S4) [16]. After isolation, the minimal functional and quantitative criteria, established by the International Society of Cell Therapy and the International Federation for Adipose Therapeutics and Science, were confirmed by flow cytometry [16, 18]. All experiments were performed in cells at P3 or P4.

### DNA methylation profiling using universal bead array

Genomic DNA was extracted from cells using the NucleoSpin® Tissue Kit (Macherery-Nagel GmbH, Dueren, Germany). DNA (cytosine) methylation profiles were generated by combining bisulfite conversion of genomic DNA and whole-genome amplification with direct, array-based capture and scoring of the CpG (cyotosine-guanine) loci. DNA samples were processed and hybridized to the Illumina EPIC array or the 850k Infinium Human-MethylationEPIC BeadChip (Illumina Inc., San Diego, CA), developed to quantitatively assay more than 850,000 methylation sites across the genome at single-nucleotide resolution, following the Infinium HD Methylation Assay Protocol. Hybridized BeadChips were imaged on an Illumina iScan system following the manufacturer’s recommendations. Gene annotation was performed using Illumina’s annotation of probes. Briefly, CpG markers present on MethylationEPIC 850k array were classified based on their chromosome location, the Infinium chemistry used to interrogate the marker (Infinium I, Infinium II), and the feature category gene region as per UCSC annotation (TSS200, TSS1500, 5′ UTR, 1st Exon, Body, 3′ UTR) [55]. Methylation data has been deposited in the GEO database with the accession code GSE138311.

### Differentially methylated loci analysis

All statistical data treatments were performed with the R statistical programming environment (version 3.5.3; R core team, 2019). DNA methylation raw data (IDAT files, Illumina iScan system direct output storing probe intensities) were read using the Bioconductor package minfi (version 1.28.3) [56, 57] and the annotation packages IlluminaHumanMethylationEPICmanifest version 3.0 and IlluminaHumanMethylationEPICanno.ilm10b2.hg19 version 6.0. Initial pre-processing and quality control were performed with minfi. The quality of each sample was assessed using the internal control probes located on the BeadChip array and samples with a *p* value > 0.01 were discarded. Probe intensities were normalized using the Stratified Quantile Normalization method [58], based on the assumption that probes with the same CpG annotation should have similar distributions. A total of 9.32% of the probes were removed as they were identified as failed in one or more samples, mapped to multiple genome sites, or associated with CpGs with known single-nucleotide polymorphisms. The CpG methylation level was calculated as *M* values (*M* = log_2_[*M*/*U*]), used for statistical analyses, and *β* values (*β* = *M*/[*M* + *U* + 100]), used for methylation level visualization [59]. DMPs were located through linear regression using dmpFinder, a minfi wraparound lmFit function of the limma software package [60].

Based on Illumina’s manifest, DMPs were assigned to genes and candidate genes were identified based on a nominal *p* value cutoff < 1 × 10^−4^ and ten or more DMPs per gene. DMRs were located using the minfi function Bumphunter [56], originally described by Jaffe et al. [29], with default settings and the smoothing option. In brief, Bumphunter looks for DMRs by searching for CpGs with a mean difference above a certain threshold. We set the inclusion threshold to 0.2. To remove non-biologically significant DMRs, we filtered for at least three consecutive CpGs. Again, significant DMRs were assigned to genes based on Illumina’s manifest, and candidate genes were selected based on *p* value cutoff < 1 × 10^−2^ and the number of DMRs per gene and their relation to the gene structure.

### Functional network analysis and visualization

For functional studies, STRING v11: protein–protein association networks (https://string-db.org/) was used to evaluate the implicated molecular and functional pathways for the candidate genes. We corroborated these enriched pathways using REVIGO (http://revigo.irb.hr/), which summarizes and visualizes lists of GO terms. Specifically, REVIGO was used to cluster significant enriched GO terms (FDR < 0.05) into similar functions.

### Isolation of human peripheral blood mononuclear cells

Human PBMCs were isolated with Ficoll-Hypaque gradients (Amersham Bioscience, Barcelona. Spain). PBMC pellets were frozen and stored at − 80 °C until DNA extraction.

### Gene expression analysis

Total RNA was isolated using the RNeasy Lipid Tissue Mini Kit (Qiagen, Hilden, Germany). RNA quantity was measured at 260 nm and purity was assessed by the OD260/OD280 ratio. One microgram of RNA was transcribed to cDNA with random primers using the Reverse Transcription System (Applied Biosystems, Foster City, CA). Quantitative gene expression was evaluated by real-time quantitative PCR (qPCR) on a 7900HT Fast Real-Time PCR System using the TaqMan Gene Expression Assay (Applied Biosystems), listed in Supplementary Table S5. Gene expression values were calculated using the comparative Ct method and expressed relative to the expression of the housekeeping gene 18S (Hs03928985_g1).

### Statistical analysis

For gene expression data, experimental results were presented as mean ± standard deviation (SD) from 10 independent experiments (independent donors), performed at least in duplicate. Comparisons between three groups were performed using nonparametric analysis of variance for multiple comparisons and Newman-Keuls correction (significance, *p* < 0.05). Statistical calculations and visualizations were performed using GraphPad Prism 6 (GraphPad Software Inc., San Diego, CA).

For clinical and anthropometrical variables, normally distributed data were expressed as mean ± SD, and for variables with no Gaussian distribution, values were expressed as median (interquartile range). Statistical analysis was performed with the Statistical Package for the Social Sciences software, version 15 (SPSS, Chicago, IL).

## Supplementary information

**Additional file 1.** Supplementary file 1.

**Additional file 2.** Supplementary file 2.

**Additional file 3.** Figure S1-S4, Table S1-S5.

## Data Availability

Authors consent to the availability of data and materials.

## Abbreviations

CD: Crohn’s disease
CF: Creeping fat
hASCs: Human adipose-derived stem cells
PBMCs: Peripheral blood mononuclear cells
IBD: Inflammatory bowel diseases
AT: Adipose tissue
SAT: Subcutaneous adipose tissue
PCA: Principal component analysis
BMI: Body mass index
FMRs: Fully methylated regions
LMRs: Low methylated regions
UMRs: Unmethylated regions
DMPs: Differentially methylated positions
DMR: Differentially methylated regions
GO: Gene ontology
TSS: Transcription start site
UTR: 5′ untranslated region
ExonBnd: Exon boundary
FDR: False discovery rate
