# Supplementary material for: Adipose stem cells from patients with Crohn’s disease show a distinctive DNA methylation pattern
Source: Clin Epigenetics. 2020 Apr 6;12:53. doi: 10.1186/s13148-020-00843-3 (PMC7137346; doi:10.1186/s13148-020-00843-3)
Supplement: Supplementary file 3 — Additional file 3. Figure S1-S4, Table S1-S5. [file 13148_2020_843_MOESM3_ESM.pdf]

Supplementary Table S1. Demographic and clinical characteristics of cohort I: methylation study in adipose-stem cells.

	Control	CD
**n**	5	7
**Sex (male/female)**	2/3	4/3
**Age (y)**	38 ± 4.6	36.1 ± 9.4
**BMI (kg/m^2)**	24.65 ± 3.6	22.9 ± 4.8
**Smoking, n (%)**	3 (60)	5 (71.4)
**Glucose (mg/dL)**	81.5 ± 5.4	77 ± 7.7
**Cholesterol (mg/dL)**	115 ± 13.5	130 ± 20.3
**HDL (mg/dL)**	39.3 ± 5.5	41.8 ± 4.6
**Triglycerides (mg/dL)**	98.7 ± 4.5	95 ± 9.4
**Insulin (µIU/mL)**	2.1 ± 0.5	7.6 ± 2.4[a]
**HOMA-IR**	0.51 ± 0.2	1.75 ± 0.55[a]
**Age of diagnosis**	-	25.1 ± 9.4
**Disease location, n (%)**		
L1 ileal	-	6 (86)
L2 colonic	-	0 (0)
L3 ileocolonic	-	1(14)
**Disease behavior,n (%)**		
B1 nonstricturing, nonpenetrating	-	1 (14)
B2 stricturing	-	3 (43)
B3 penetraiting	-	3 (43)
**Immunomodulator use**	-	7/7
**Biological agent treatment**	-	1/7
**Steroid treatment**	-	6/7
**C-reactive protein (mg/dL)**	0.3 ± 0.10	3.3 ± 1.3[a]
**Fecal calprotectin (µg/g)**	-	1560 ± 230[a]

Abbreviations: CD; Crohn's Disease; BMI, body mass index; HDL, high-density lipoprotein cholesterol; HOMA-IR, insulin resistance index.
Results are presented as mean ± SD. ANOVA followed by *post hoc* Bonferroni test was used to compare means between groups.
[a]*p* < 0.01 *versus* control-adipose stem cells.

Supplementary Table S2. Demographic and clinical characteristics of cohort II: gene expression study in adipose-stem cells.

	Control	Inactive CD	Active CD
**n**	10	10	10
**Sex (male/female)**	4/6	5/5	5/5
**Age (years)**	40 ± 5.3	38.2 ± 3.4	36.8 ± 10.4
**BMI (kg/m^2)**	25.15 ± 2.1	24.59 ± 3.3	23.3 ± 3.3
**Smoking, n (%)**	5 (50)	6 (60)	5 (50)
**Glucose (mg/dL)**	81.3 ± 3.9	86 ± 11.4	75 ± 10.7
**Cholesterol (mg/dL)**	115 ± 12.3	121 ± 20.4	141 ± 16.3[a]
**HDL (mg/dL)**	36.7 ± 5.6	44.7 ± 3.2[a]	41.4 ± 5.4
**Triglycerides (mg/dL)**	100 ± 5.3	116 ± 13.1	95 ± 8.9
**Insulin (µIU/mL)**	2.07 ± 0.9	5.05 ± 1.2	7.5 ± 1.4[a,b]
**HOMA-IR**	0.49 ± 0.1	1.3 ± 0.5	1.65 ± 0.35[a]
**Age at diagnosis (years)**	-	38 ± 8.6	27.9 ± 9.4
**Last attack (months)**		21± 7.4	
**Disease location, n (%)**			
L1 ileal	-	6/10 (60)	7/10 (70)
L2 colonic	-	3/10 (30)	2/10 (20)
L3 ileocolonic	-	2/10 (20)	1/10 (10)
**Disease behavior,n (%)**			
B1 nonstricturing, nonpenetrating	-	0/10 (0)	0/10 (0)
B2 stricturing	-	5/10 (50)	4/10 (40)
B3 penetraiting	-	5/10 (50)	6/10 (60)
**Indication of surgery**	CoH	CoH	4SCD/6FCD
**Immunomodulator use**	-	10/10	10/10
**Biological agent treatment**	-	2/10	2/10
**Steroid treatment**	-	4/10	8/10
**C-reactive protein (mg/dL)**	0.2 ± 0.04	0.3 ± 0.13	3.4 ± 1.6[a,b]
**Fecal calprotectin (µg/g)**	-	81.25 ± 20.3	2132.4 ± 159.1[b]

Abbreviations: BMI, body mass index; HDL, high-density lipoprotein cholesterol; CoH, cholecystectomy or hernia; CD; Crohn's Disease; SCD, stenotic Crohn's disease; FCD, fistulizing Crohn's disease.
Results are presented as mean ± SD. ANOVA followed by *post hoc* Bonferroni test was used to compare means between groups.
[a]$p < 0.01$ *versus* control-adipose stem cells.
[b]$p < 0.05$ *versus* inactive CD-adipose stem cells.

Supplementary Table S3. Demographic and clinical characteristics of cohort III: gene expression study in peripheral blood mononuclear cells.

	Control	Inactive CD	Active CD
**n**	10	10	10
**Sex (male/female)**	5/5	5/5	5/5
**Age (y)**	39.3 ± 6.9	38.8 ± 14.14	45.14 ± 18.14
**BMI (kg/m^2)**	24.4 ± 1.4	25.52 ± 5.22	23.06 ± 3.37
**Smoking, n (%)**			
Active	3 (30)	5 (50)	3 (30)
Ex	3 (30)	2 (20)	3 (30)
No	4 (40)	3 (30)	4 (40)
**Glucose (mg/dL)**	75 ± 3.9	86.6 ± 10.13[a]	84.33 ± 11.29[a]
**Cholesterol (mg/dL)**	139 ± 11.3	198 ± 52.09[a]	164.75 ± 22.8[a]
**Cholesterol-HDL (mg/dL)**	60.61 ± 20.15	64.22 ± 22.52	42 ± 15.55[a,b]
**Triglycerides**	123 ± 35.6	141 ± 43.8	106 ± 25.45
**Age of diagnosis**	-	19 ± 4.7	22 ± 5.8
**Last attack (months)**	-	20 ± 3.8	-
**Disease location, n (%)**			
L1 ileal	-	8 (80)	10 (100)
L2 colonic	-	1 (10)	0 (0)
L3 ileocolonic	-	1 (10)	0 (0)
**Disease behavior, n (%)**			
B1 nonstricturing, nonpenetrating	-	6 (60)	4 (40)
B2 stricturing	-	3 (30)	3 (30)
B3 penetraiting	-	1 (10)	3 (30)
**Immunomodulator use**	-	8/10	7/10
**Biological agent treatment**	-	7/10	6/10
**Steroid treatment**	-	4/10	4/10
**C-reactive protein (mg/dL)**	0.14 ± 0.04	0.21 ± 0.07	3.4 ± 1.1[a,b]
**Fecal calprotectin (µg/g)**	-	178.75 ± 23.4	2219 ± 117.15[b]

Abbreviations: BMI, body mass index; HDL, high-density lipoprotein cholesterol; CoH, cholecystectomy or hernia; CD; Crohn's Disease.
Results are presented as mean ± SD. ANOVA followed by *post hoc* Bonferroni test was used to compare means between groups.
[a]$p < 0.01$ *versus* control-peripheral mononuclear cells (PBMCs).
[b]$p < 0.01$ *versus* inactive CD-PBMCs.

Supplementary Table S4. Immunophenotypic characterization of hASCs isolated from healthy control subjects, active CD and inactive CD patients. The values reflect the mean ±SD percentage surface positive staining of hASCs for a part of surface antigens including hematopoietic and stromal markers.

	Control		Inactive CD		Active CD	
**Surface markers**	%	MFI	%	MFI	%	MFI
CD34	0.2±0.1	80±9.2	0.91±0.4	125±10.7[a]	0.9±0.5	200±16.4[a]
CD45	0.3±0.25	110±10.4	0.3±0.19	87±8.3	0.2±0.15	90±9.5
CD90	95.3±2.1	18800±725.2	97.3±2.4	22400±888	97.9±2	31000±1025
CD73	90±7.2	9880±200	86.5±6.6	8400±176	87.5±8.1	5900±110[a]
CD36	40±10.3	700±25	32.5±10.3	190±30[a]	30.5±10.3	200±45[a]

Abbreviations: hASCs, human adipose derived stem cells; MFI, median fluorescence intensity.
ANOVA followed by post hoc Bonferroni test was used to compare means between groups. * $P < 0.05$ *versus* Control-hASCs.

Gene name	Detector
**C2**	Hs00918862_m1
**CHID1**	Hs01061050_m1
**DLX5**	Hs00193291_m1
**EBF3**	Hs01008793_m1
**EN1**	Hs00154977_m1
**FGF1**	Hs01092738_m1
**FGF13**	Hs01381548_m1
**FGFR2**	Hs01552918_m1
**G6PD**	Hs00166169_m1
**HOXB5**	Hs00357820_m1
**HOXB6**	Hs00980016_m1
**IKBKE**	Hs01063858_m1
**IKBKG**	Hs00415849_m1
**LTBR**	Hs01101194_m1
**NOTCH4**	Hs00965889_m1
**PRDM8**	Hs00220274_m1
**PRDM16**	Hs00223161_m1
**PTPRN2**	Hs00243067_m1
**RIPK1**	Hs01041869_m1
**SHROOM3**	Hs01068190_m1
**SPON2**	Hs00202813_m1
**TNF**	Hs00174128_m1
**WT1**	Hs01103751_m1

Supplemental Table S5. Human gene expression analysis. Results were calculated using the comparative Ct method and expressed relative to the expression of the housekeeping gene 18S (Hs03928985_g1).

REVIGO

Biological Process (131) | Tag Clouds

Scatterplot & Table | Interactive Graph | TreeMap

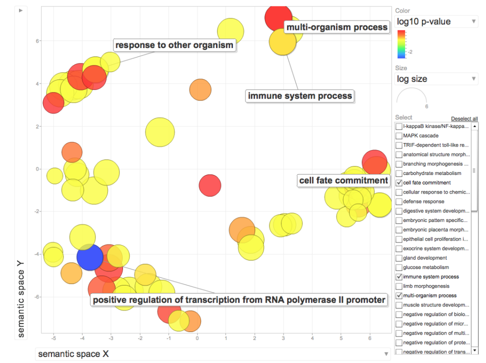

click empty space and and drag to zoom

interpretation of the coordinate axes?

While parsing your data, warning(s) were encountered:
Could not parse GO ID from line: '#term ID false discovery rate'. Line will be skipped.
Go term 62013 was not found in the current version of the GeneOntology, dated 22:12:2016 16:59. GO term will be skipped.
Go term 110110 was not found in the current version of the GeneOntology, dated 22:12:2016 16:59. GO term will be skipped.

Tip: your resulting list of GO terms seems to be quite long. If you want to reduce it further, press the Back button in your browser and choose a different setting for the "allowed similarity" parameter.

Hide/show dispensable GO terms | Export results to text table (CSV) | Make R script for plotting

term ID	description	frequency	pin?	log_{10} p-value	uniqueness	dispensability
GO:0002376	immune system process	0.600 %		-1.5986	0.98	0.00
GO:0045944	positive regulation of transcription from RNA polymerase II promoter	0.369 %		-2.2076	0.58	0.00
GO:0001934	*positive regulation of protein phosphorylation*	0.227 %		-1.6402	0.58	0.78
GO:0048522	*positive regulation of cellular process*	1.585 %		-1.6402	0.58	0.91
GO:0031325	*positive regulation of cellular metabolic process*	1.004 %		-1.6402	0.57	0.98
GO:0031347	*regulation of defense response*	0.132 %		-1.6402	0.59	0.74
GO:0048584	*positive regulation of response to stimulus*	0.461 %		-1.6402	0.49	0.72
GO:0051254	*positive regulation of RNA metabolic process*	0.535 %		-1.6402	0.58	0.94
GO:0006357	*regulation of transcription from RNA polymerase II promoter*	1.273 %		-1.6402	0.68	0.84
GO:0071902	*positive regulation of protein serine/threonine kinase activity*	0.075 %		-1.6383	0.60	0.92
GO:0043410	*positive regulation of MAPK cascade*	0.111 %		-1.6402	0.45	0.93
GO:0032101	*regulation of response to external stimulus*	0.160 %		-1.3605	0.59	0.75
GO:1902533	*positive regulation of intracellular signal transduction*	0.214 %		-1.6402	0.47	0.77
GO:0010604	*positive regulation of macromolecule metabolic process*	0.988 %		-1.6402	0.57	0.88
GO:0032147	*activation of protein kinase activity*	0.081 %		-1.6402	0.59	0.91
GO:0043123	*positive regulation of I-kappaB kinase/NF-kappaB signaling*	0.037 %		-1.6402	0.52	0.81
GO:0050727	*regulation of inflammatory response*	0.051 %		-1.6402	0.60	0.80
GO:0009966	*regulation of signal transduction*	0.857 %		-1.4401	0.52	0.88
GO:0080134	*regulation of response to stress*	0.337 %		-1.5157	0.58	0.80
GO:0000187	*activation of MAPK activity*	0.024 %		-1.6402	0.49	0.83
GO:0051173	*positive regulation of nitrogen compound metabolic process*	0.685 %		-1.6402	0.58	0.94
GO:0051704	multi-organism process	0.751 %		-1.3605	0.98	0.00
GO:0051707	response to other organism	0.299 %		-1.6402	0.80	0.00
GO:0009615	*response to virus*	0.117 %		-1.6402	0.81	0.92
GO:0032496	*response to lipopolysaccharide*	0.043 %		-1.6402	0.79	0.85
GO:0060669	embryonic placenta morphogenesis	0.004 %		-1.6402	0.62	0.00
GO:0006006	glucose metabolic process	0.430 %		-1.6402	0.90	0.05
GO:0005975	carbohydrate metabolic process	5.260 %		-1.6402	0.97	0.07
GO:0006468	protein phosphorylation	4.137 %		-1.6402	0.89	0.16
GO:0010883	regulation of lipid storage	0.010 %		-1.5986	0.80	0.18
GO:0031111	negative regulation of microtubule polymerization or depolymerization	0.007 %		-1.6402	0.72	0.18
GO:0040008	regulation of growth	0.172 %		-1.6402	0.70	0.22
GO:0060420	*regulation of heart growth*	0.008 %		-1.6402	0.46	0.73
GO:0060421	*positive regulation of heart growth*	0.005 %		-1.6402	0.40	0.94
GO:0055021	*regulation of cardiac muscle tissue growth*	0.007 %		-1.4895	0.45	0.97
GO:0045926	*negative regulation of growth*	0.052 %		-1.6402	0.67	0.87
GO:0048638	*regulation of developmental growth*	0.085 %		-1.6402	0.47	0.85
GO:0048639	*positive regulation of developmental growth*	0.039 %		-1.5952	0.40	0.82
GO:0040014	*regulation of multicellular organism growth*	0.023 %		-1.3439	0.46	0.87
GO:0030308	*negative regulation of cell growth*	0.030 %		-1.5986	0.64	0.79
GO:0002682	regulation of immune system process	0.252 %		-1.6402	0.68	0.23
GO:0030099	*myeloid cell differentiation*	0.074 %		-1.4461	0.48	0.77
GO:0002684	*positive regulation of immune system process*	0.165 %		-1.6108	0.57	0.92
GO:0045087	*innate immune response*	0.148 %		-1.6402	0.73	0.81
GO:0050778	*positive regulation of immune response*	0.112 %		-1.6180	0.48	0.85
GO:0050776	*regulation of immune response*	0.150 %		-1.6198	0.56	0.90
GO:0006955	*immune response*	0.337 %		-1.6126	0.74	0.87
GO:0042981	regulation of apoptotic process	0.313 %		-1.6402	0.69	0.24
GO:0010941	*regulation of cell death*	0.344 %		-1.6402	0.69	0.94
GO:0060548	*negative regulation of cell death*	0.191 %		-1.5186	0.65	0.94
GO:0008630	*intrinsic apoptotic signaling pathway in response to DNA damage*	0.021 %		-1.3325	0.64	0.77
GO:0007249	I-kappaB kinase/NF-kappaB signaling	0.057 %		-1.6402	0.66	0.28
GO:0048519	negative regulation of biological process	1.984 %		-1.6402	0.72	0.28
GO:0036092	phosphatidylinositol-3-phosphate biosynthetic process	0.011 %		-1.5171	0.88	0.29
GO:0010469	regulation of receptor activity	0.025 %		-1.6402	0.60	0.32
GO:0048518	positive regulation of biological process	1.744 %		-1.6402	0.73	0.33
GO:0051246	regulation of protein metabolic process	1.551 %		-1.3575	0.69	0.39
GO:0042221	response to chemical	3.071 %		-1.6402	0.79	0.40
GO:0006952	defense response	0.568 %		-1.6402	0.80	0.43
GO:0060664	epithelial cell proliferation involved in salivary gland morphogenesis	0.001 %		-1.6402	0.64	0.44
GO:0048754	branching morphogenesis of an epithelial tube	0.035 %		-1.6402	0.57	0.45
GO:0060688	*regulation of morphogenesis of a branching structure*	0.012 %		-1.5003	0.50	0.90
GO:0048566	*embryonic digestive tract development*	0.008 %		-1.6402	0.60	0.73
GO:1905330	*regulation of morphogenesis of an epithelium*	0.026 %		-1.6402	0.52	0.80
GO:0060602	*branch elongation of an epithelium*	0.004 %		-1.6402	0.59	0.86
GO:0001658	*branching involved in ureteric bud morphogenesis*	0.012 %		-1.5654	0.57	0.97
GO:0001657	*ureteric bud development*	0.020 %		-1.6402	0.58	0.77
GO:0070887	cellular response to chemical stimulus	1.007 %		-1.6402	0.76	0.46
GO:0071310	*cellular response to organic substance*	0.651 %		-1.6402	0.76	0.70
GO:0010033	*response to organic substance*	0.900 %		-1.6402	0.78	0.73
GO:0035456	response to interferon-beta	0.004 %		-1.6402	0.83	0.46
GO:0048583	regulation of response to stimulus	1.120 %		-1.6402	0.64	0.46
GO:0009605	response to external stimulus	1.370 %		-1.6402	0.80	0.47
GO:0045667	regulation of osteoblast differentiation	0.021 %		-1.6402	0.49	0.48
GO:0006970	response to osmotic stress	0.082 %		-1.3516	0.82	0.48
GO:0035272	exocrine system development	0.022 %		-1.6402	0.61	0.50
GO:0035108	limb morphogenesis	0.030 %		-1.6402	0.61	0.51
GO:0055057	neuroblast division	0.005 %		-1.6402	0.61	0.51
GO:0000165	MAPK cascade	0.245 %		-1.6402	0.60	0.51
GO:0001503	ossification	0.074 %		-1.6402	0.66	0.52
GO:0045892	negative regulation of transcription, DNA-templated	0.592 %		-1.6402	0.65	0.54
GO:0051253	*negative regulation of RNA metabolic process*	0.626 %		-1.6402	0.65	0.97
GO:0000122	*negative regulation of transcription from RNA polymerase II promoter*	0.199 %		-1.6402	0.68	0.89
GO:0051172	*negative regulation of nitrogen compound metabolic process*	0.792 %		-1.3019	0.66	0.87
GO:0045071	negative regulation of viral genome replication	0.008 %		-1.5114	0.76	0.54
GO:0050766	positive regulation of phagocytosis	0.009 %		-1.5498	0.66	0.55
GO:0035666	TRIF-dependent toll-like receptor signaling pathway	0.000 %		-1.6402	0.58	0.55
GO:0006950	response to stress	4.575 %		-1.6402	0.78	0.56
GO:0048732	gland development	0.095 %		-1.6402	0.56	0.56
GO:0021879	*forebrain neuron differentiation*	0.011 %		-1.5229	0.58	0.83
GO:0030901	*midbrain development*	0.019 %		-1.5560	0.59	0.86
GO:0030900	*forebrain development*	0.071 %		-1.5670	0.56	0.94
GO:0007420	*brain development*	0.142 %		-1.5186	0.54	0.71
GO:0031399	regulation of protein modification process	0.565 %		-1.4306	0.68	0.57
GO:0051897	positive regulation of protein kinase B signaling	0.016 %		-1.6402	0.54	0.57
GO:0045165	cell fate commitment	0.091 %		-1.6402	0.62	0.57
GO:0055123	digestive system development	0.039 %		-1.6402	0.59	0.58
GO:0051781	positive regulation of cell division	0.023 %		-1.6402	0.65	0.58
GO:0003156	regulation of animal organ formation	0.008 %		-1.6402	0.50	0.59
GO:0060541	respiratory system development	0.054 %		-1.5143	0.59	0.60
GO:0051239	regulation of multicellular organismal process	0.628 %		-1.6402	0.49	0.61
GO:0043085	positive regulation of catalytic activity	0.818 %		-1.4101	0.72	0.61
GO:0010893	positive regulation of steroid biosynthetic process	0.003 %		-1.6402	0.67	0.62
GO:0009880	embryonic pattern specification	0.025 %		-1.6402	0.60	0.63
GO:0048568	*embryonic organ development*	0.113 %		-1.6402	0.55	0.87
GO:0048598	*embryonic morphogenesis*	0.171 %		-1.6402	0.56	0.79
GO:1902932	positive regulation of alcohol biosynthetic process	0.004 %		-1.6402	0.67	0.63
GO:0009653	anatomical structure morphogenesis	1.542 %		-1.6402	0.60	0.64
GO:0048513	*animal organ development*	0.779 %		-1.5200	0.51	0.79
GO:0030154	*cell differentiation*	1.133 %		-1.6402	0.56	0.80
GO:0009790	*embryo development*	0.311 %		-1.6402	0.55	0.72
GO:0050793	*regulation of developmental process*	1.205 %		-1.5654	0.45	0.81
GO:0007275	*multicellular organism development*	1.559 %		-1.3170	0.51	0.92
GO:0009887	*animal organ morphogenesis*	0.264 %		-1.6383	0.53	0.80
GO:0048731	*system development*	1.264 %		-1.3439	0.51	0.89
GO:0016202	regulation of striated muscle tissue development	0.023 %		-1.6402	0.46	0.65
GO:0045843	*negative regulation of striated muscle tissue development*	0.008 %		-1.5654	0.45	0.94
GO:0051147	*regulation of muscle cell differentiation*	0.033 %		-1.6402	0.51	0.80
GO:0001501	skeletal system development	0.107 %		-1.6402	0.57	0.65
GO:0010803	regulation of tumor necrosis factor-mediated signaling pathway	0.005 %		-1.4698	0.62	0.66
GO:0061061	muscle structure development	0.142 %		-1.3516	0.63	0.67
GO:0007389	pattern specification process	0.147 %		-1.4776	0.57	0.67
GO:0002718	regulation of cytokine production involved in immune response	0.013 %		-1.3439	0.40	0.68
GO:0002920	*regulation of humoral immune response*	0.009 %		-1.3401	0.62	0.71
GO:0032755	*positive regulation of interleukin-6 production*	0.013 %		-1.3152	0.46	0.76
GO:0034340	response to type I interferon	0.008 %		-1.3401	0.74	0.68
GO:1901700	response to oxygen-containing compound	0.503 %		-1.3526	0.79	0.68
GO:0051094	positive regulation of developmental process	0.258 %		-1.5229	0.39	0.69
GO:0051241	negative regulation of multicellular organismal process	0.211 %		-1.6402	0.44	0.69
GO:2000027	*regulation of organ morphogenesis*	0.044 %		-1.6402	0.45	0.72
GO:0043242	negative regulation of protein complex disassembly	0.041 %		-1.3809	0.70	0.69

Did you find REVIGO useful in your work? Feel free to share it with your colleagues.

Like 201 | Tweet

Supplementary Figure S1. Functional analysis and visualization of genes up-regulated in adipose-stem cells isolated of CD patients. The enriched pathways were visualized and the network analysis performed using REVIGO (https:// http://revigo.irb.hr/), which summarizes and visualizes lists of gene ontology [GO] terms.

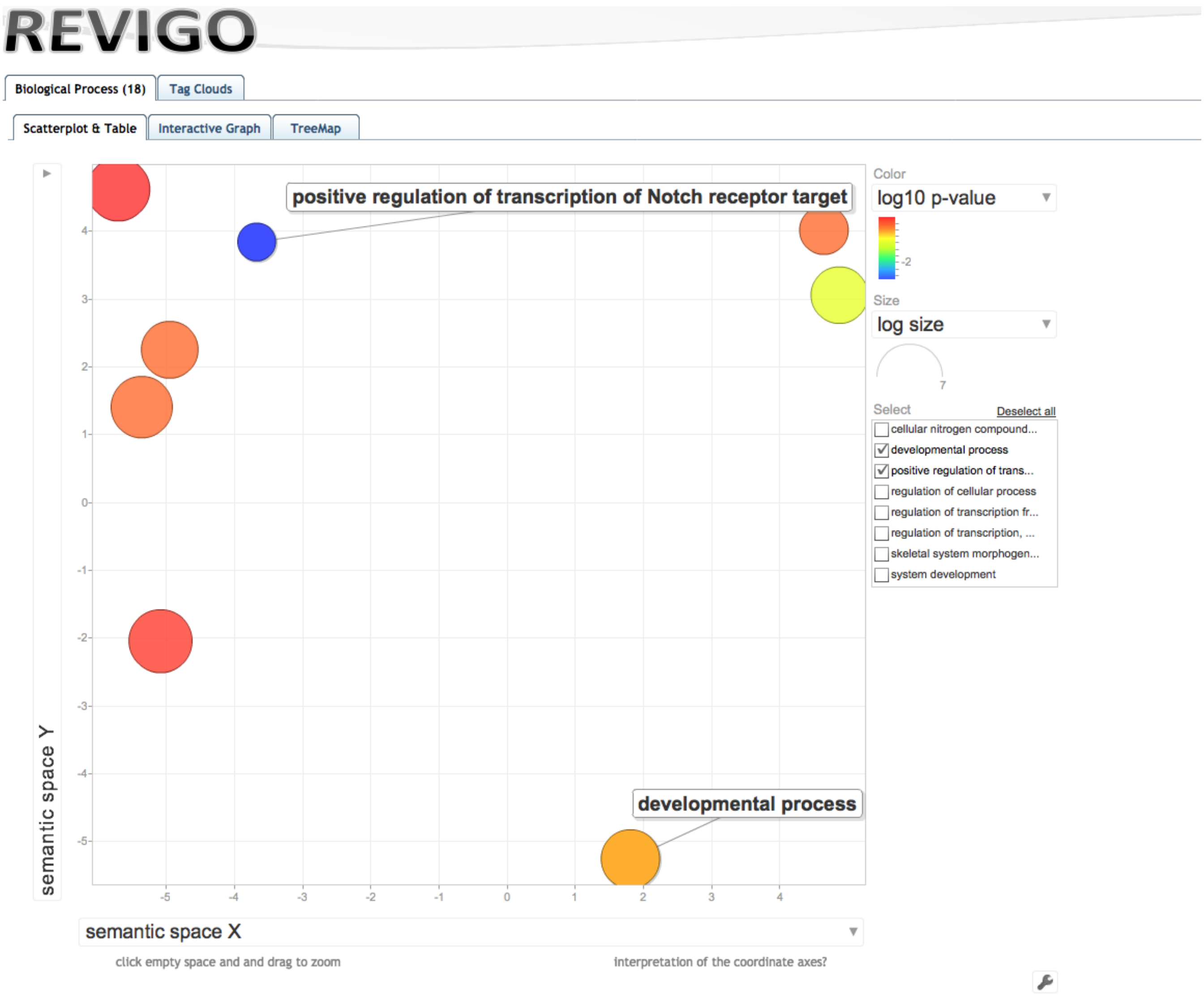

While parsing your data, warning(s) were encountered:
Could not parse GO ID from line: '#term ID false discovery rate'. Line will be skipped.

Hide/show dispensable GO terms · Export results to text table (CSV) · Make R script for plotting

term ID	description	frequency	pin?	log_{10} p-value	uniqueness	dispensability
GO:0007221	positive regulation of transcription of Notch receptor target	0.002 %		-2.2596	0.58	0.00
GO:0032502	developmental process	2.812 %		-1.5243	0.89	0.00
GO:0048731	system development	1.264 %		-1.6799	0.43	0.05
GO:0009653	*anatomical structure morphogenesis*	1.542 %		-1.3125	0.46	0.84
GO:0007389	*pattern specification process*	0.147 %		-1.4271	0.49	0.75
GO:0030154	*cell differentiation*	1.133 %		-1.4271	0.43	0.79
GO:0007399	*nervous system development*	0.582 %		-1.4271	0.45	0.86
GO:0007275	*multicellular organism development*	1.559 %		-1.6615	0.43	0.92
GO:0044271	cellular nitrogen compound biosynthetic process	22.502 %		-1.3363	0.59	0.13
GO:0050794	regulation of cellular process	18.840 %		-1.3054	0.49	0.21
GO:0006357	regulation of transcription from RNA polymerase II promoter	1.273 %		-1.4271	0.42	0.55
GO:0006355	regulation of transcription, DNA-templated	9.917 %		-1.4271	0.32	0.66
GO:0019219	*regulation of nucleobase-containing compound metabolic process*	10.258 %		-1.4271	0.37	0.86
GO:0031326	*regulation of cellular biosynthetic process*	10.816 %		-1.4271	0.36	0.89
GO:0051252	*regulation of RNA metabolic process*	10.029 %		-1.3696	0.35	0.89
GO:0006351	*transcription, DNA-templated*	10.659 %		-1.4271	0.47	0.88
GO:2000112	*regulation of cellular macromolecule biosynthetic process*	10.683 %		-1.4271	0.35	0.88
GO:0048705	skeletal system morphogenesis	0.053 %		-1.4271	0.51	0.69

Did you find REVIGO useful in your work? Feel free to share it with your colleagues.

Like 201 Tweet

Supplementary Figure S2. Functional analysis and visualization of genes down-regulated in adipose-stem cells isolated of CD patients. The enriched pathways were visualized and the network analysis performed using REVIGO (https:// http://revigo.irb.hr/), which summarizes and visualizes lists of gene ontology [GO] terms.

A. *Immune system*

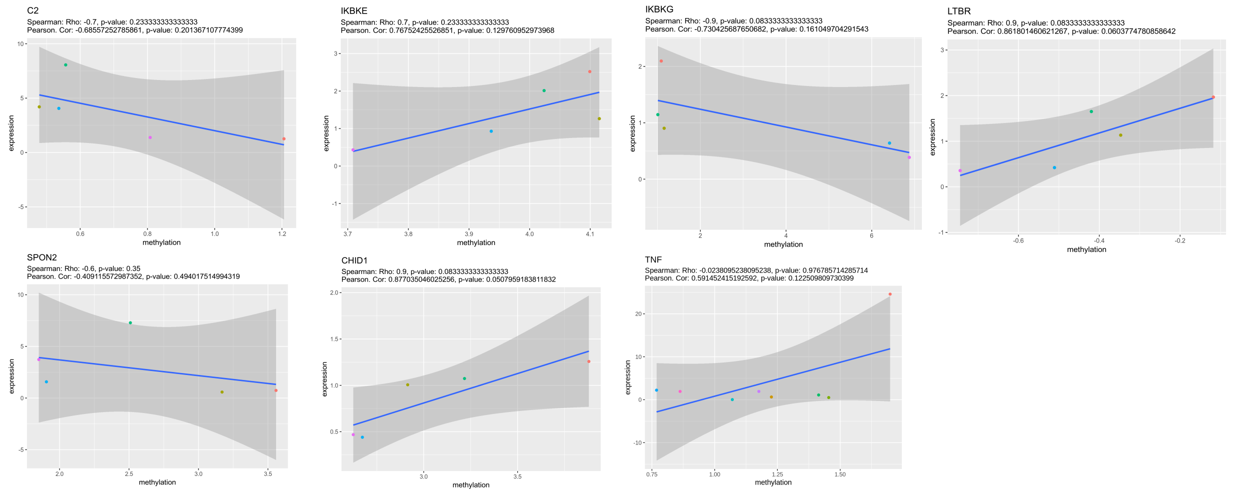

B. *Metabolic process*

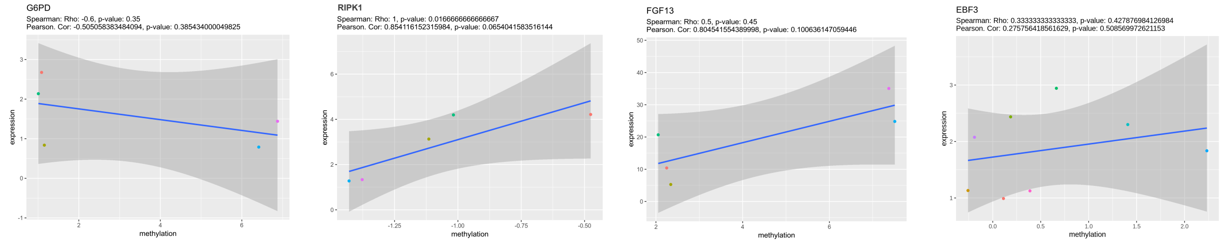

C. *Cell Differentiation*

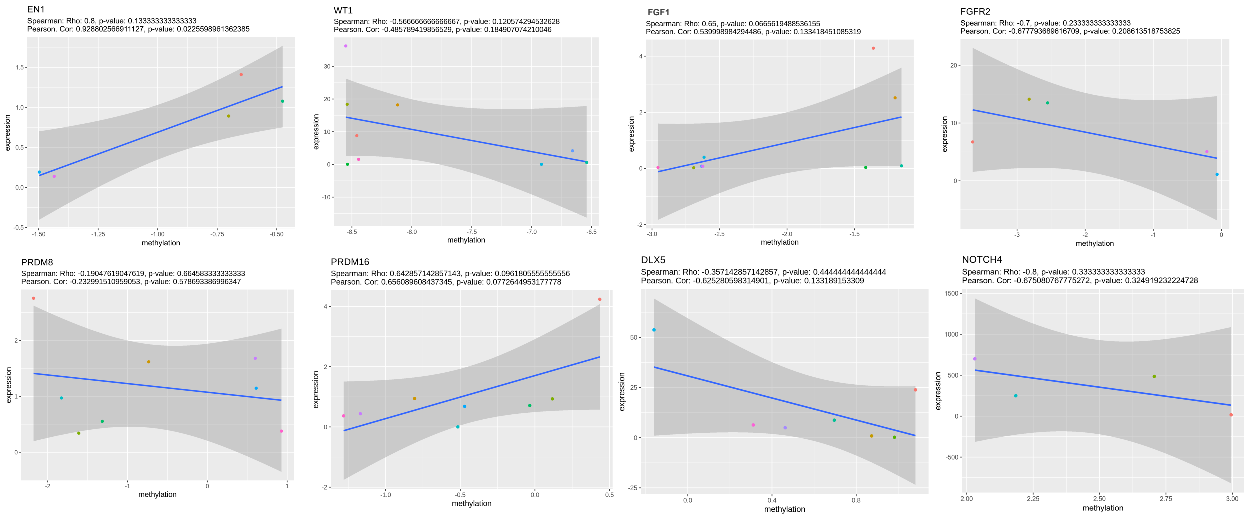

D. *Development process*

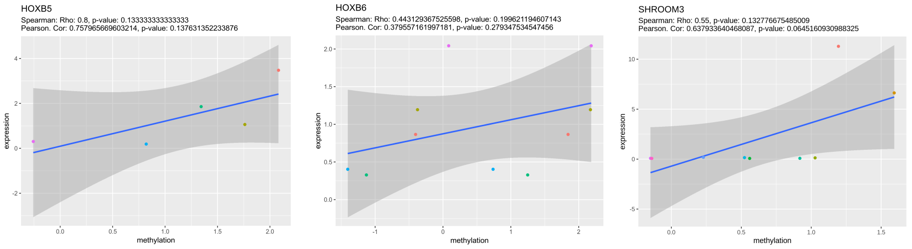

Supplementary Figure S3. Pearson and Spearman correlations between gene expression and methylation data of candidate genes obtained from differentially methylated regions. (A) Genes involved in immune system response: *C2, IKBKE, IKBKG, LTBR, SPON2, CHID1* and *TNFA*. (B) Genes related to the regulation of metabolism: *G6PD, FGF13, EBF3, RIPK1*. (C) Genes related to cell differentiation: *EN1, WT1, FGF1, FGFR2, PRDM8, PRDM16* and *DLX5.*

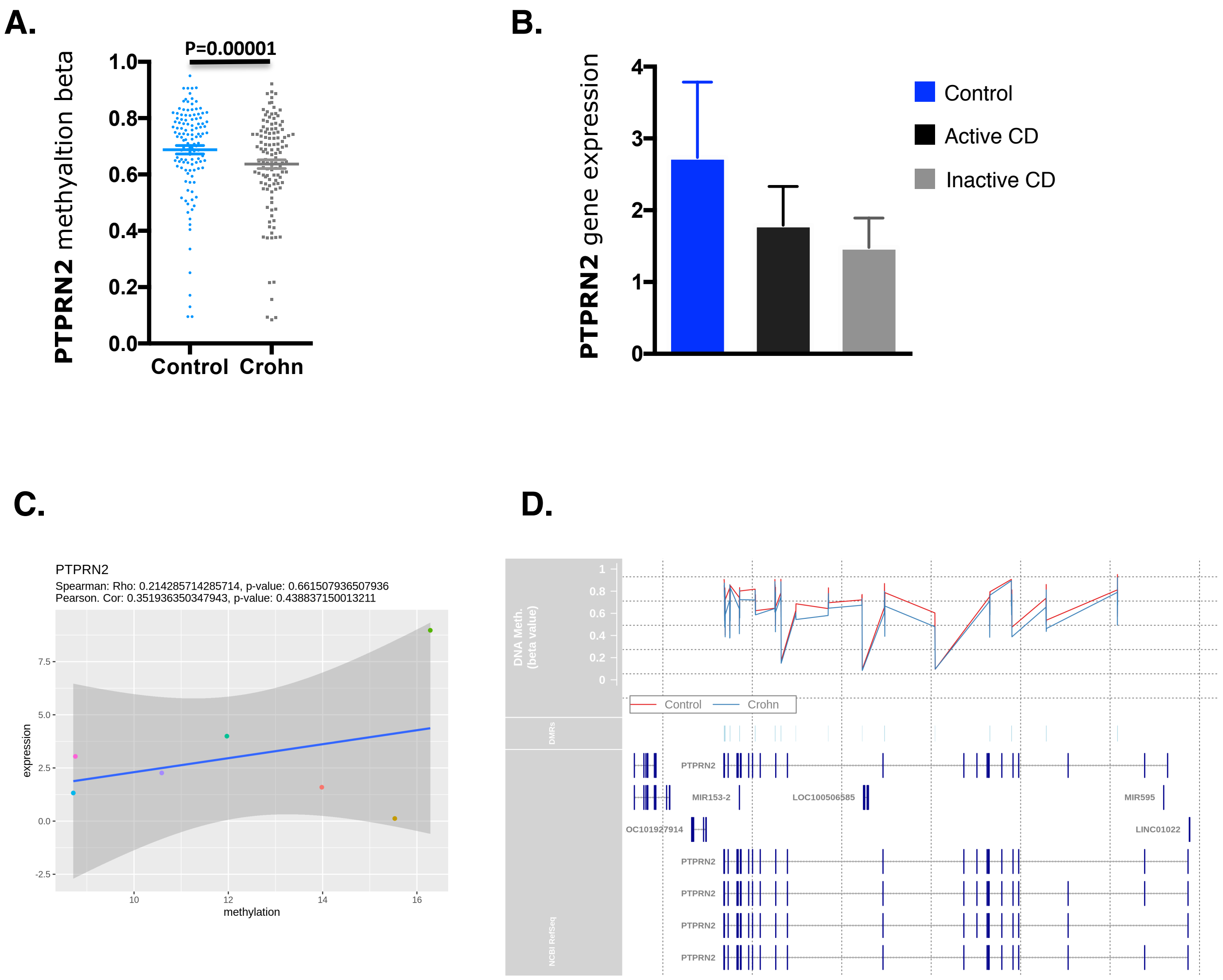

Supplementary Figure S4. *PTPNR2* is hypomethylated in human adipose stem cells of patients with Crohn's disease but there is no correlation with gene expression. Methylation (a) and mRNA levels (b) of *PTPNR2* in hASCs. (c) Spearman and Person's correlation between methylation and gene expression data. (d) DMR plot in *PTPNR2* gene.
